# Function of Trunk-Mediated “Greeting” Behaviours between Male African Elephants: Insights from Choice of Partners

**DOI:** 10.3390/ani11092718

**Published:** 2021-09-17

**Authors:** Connie R. B. Allen, Darren P. Croft, Camille Testard, Lauren J. N. Brent

**Affiliations:** 1 Centre for Research in Animal Behaviour, College of Life and Environmental Sciences, University of Exeter, Exeter EX4 4QG, UK; d.p.croft@exeter.ac.uk (D.P.C.); l.j.n.brent@exeter.ac.uk (L.J.N.B.); 2 Elephants for Africa, 5 Balfour Road, London N5 2HB, UK; 3 School of Biological Sciences, University of Bristol, Life Sciences Building, 24 Tyndall Avenue, Bristol BS8 1TQ, UK; 4 Department of Neuroscience, University of Pennsylvania, Philadelphia, PA 19104, USA; camille.testard@pennmedicine.upenn.edu

**Keywords:** male-male communication, olfactory assessment, greetings, elephants, long-lived mammals, tactile communication

## Abstract

**Simple Summary:**

Despite our common connotations with the term “greeting”, such behaviours in non-human societies are not exclusively employed in the context of reunion. For example, these multimodal signals can function to test bonds, promote cooperation, or facilitate further positive interactions between individuals, as well as to ease tension and update uncertain relationships at reunion events. A common behaviour in male elephants is for a male to direct his trunk to a same-sex conspecific’s mouth, temporal gland, or genitals. Such behaviours are often labelled as greetings, and in addition to being gestural and tactile signals, may simultaneously enable olfactory assessment of aspects of the target male’s phenotype. We analysed trunk-mediated greetings performed by different aged male elephants at aggregations in an all-male area. Despite considerable mixing of new potential interactors, in a system with high fission-fusion dynamics, we found no evidence that males preferentially targeted elephants met new at aggregations with greetings. Adolescents greeted at higher rates than adults. All males, apart from older adolescents preferably greeted age-matched males. In male-male elephant communication, these behaviours likely function to facilitate further positive interaction, or assess aspects of phenotype between similar aged males occupying the same ecological space, rather than as a reunion display.

**Abstract:**

A common behavioural interaction between male African elephants is for an actor to direct his trunk to contact a same sex conspecific’s mouth, temporal gland, or genital region. Such behaviours are often referred to as “greetings”. Along with its inherent tactile element, these behaviours also likely provide olfactory information to actors concerning aspects of the target’s phenotype, including sexual status, feeding history, individual identity, and emotional state. Here, we explore whether the age and novelty of potential interactors affect the choice of individuals targeted by male African elephants for these trunks to scent emitting organ (SEO) behaviours at social hotspots in a male-dominated area. Male elephants of all ages, except older adolescents aged 16–20 years, preferentially targeted elephants of the same age class for trunk-to-SEO behaviours. Elephants younger than 26 years did not direct trunk-to-SEO behaviours to mature bulls (26+ years) more than expected by chance, suggesting these behaviours are not primarily used for younger males to establish contact with, or obtain information from or about older, more experienced individuals. We also found no evidence that males directed these behaviours preferentially to new individuals they encountered at male aggregations (compared to those they arrived in groups with), suggesting these behaviours are not primarily employed by males as a reunion display to establish relationships between new individuals or update relationships between familiar individuals separated over time. Age-mates may be preferentially targeted with these behaviours as a means to facilitate further interaction with partners (e.g., for sparring activity), or as a safe way to assess relative dominance rank in similarly aged and hence, size and strength, matched dyads. Our results suggest male African elephants use close contact trunk-to-SEO behaviours continuously over time, to facilitate positive relationships, test willingness to interact, and assess aspects of phenotype, between males occupying the same ecological space.

## 1. Introduction

Chemical products released by animals play an important role in communication in animal societies [1,2]. Often, owing to the ability for chemical cues and signals to remain long after the depositor has departed, such communication can occur remotely between individuals, for example, in scent marking of territory [3,4] and sexual advertising [5]. However, many species engage in close contact olfactory assessments of conspecifics which, considering the potential risks of close proximity, even tactile contact between individuals, may also overlap with other social messages; tests of dominance, relationship strength, or willingness to interact [6,7,8,9]. Sniffing behaviours in rats, for example, are not solely used for obtaining olfactory information, but also to convey appeasement during social interactions [10]. Such tactile behaviours are observed in elephant species, whereby close physical contact is initiated by the subject directing its trunk towards a target elephant’s mouth, temporal gland or genitals [11]. These behaviours are performed in a diverse range of contexts, including during reunion, social play, conciliation, and coalition building [12]. The mouth, temporal glands and genitals of elephants are known to emit chemical products, so it is likely an element of olfactory assessment can also be conducted by the actor [13]. The secretions of these organs in elephants may encode various aspects of phenotype, such as sexual status [14,15], feeding history [16,17], individual identity [18], and potentially age and emotional state [11,19]. The adaptive function of these trunk behaviours between males is particularly interesting because it involves risky close contact between potential competitors [20], as well as a risk of disease transmission owing to close or direct contact of body orifices [21]. This study aims to provide new insights into the function of trunk to scent emitting organ (SEO) behaviours in male African Savannah elephants (*Loxodonta africana*, hereby referred to as “African elephants”), particularly concerning their occurrence between partners of similar or divergent age classes in this long-lived mammal.

Male African elephants live in societies with a high degree of fission-fusion dynamics, whereby individuals maintain diverse and loose associations with other males of mixed age and level of maturity [22,23]. This is in contrast to the more tightly bonded groups of females that are primarily held together by strong kinship ties, within higher levels of social organisation [24,25]. Outside of sexually active “musth” periods, males are generally gregarious [14,26], spending around 63% of their time in all-male groups [23]. Male African elephants have an adolescent life history stage between the ages of 10–20 years [27,28], and full sexual maturity, and first paternity is in general not achieved till the age of 25–30 years [27,29,30]. There is evidence that males can hold stable relationships over time, although the long-term time scale for these relationships is not yet clear [27,31]. Whilst a number of studies have looked at the patterns of social associations within male African elephant society [23,26,31], the specific behavioural interactions between males in all-male groups has less often been the focus of research (but on aggression see [14,32], and on sparring [23,28]).

In male African elephant communication, the trunk-to-SEO behaviours described are often labelled as “greetings” [11,12]. Greeting behaviours in other species similarly can involve an aspect of inspection of sources of scent (e.g., armpit and genital sniffing in howler monkeys, *Alouatta palliata*, [8]; inspection of anogenital region in wolves, *Canis lupus,* [33] and spotted hyenas, *Crocuta crocuta,* [34]), as well as intimate tactile contact (e.g., genital fondling in baboons, *Papio* sp. [35]; embraces in spider monkeys, *Ateles geoffroyi* [36]). Greeting behaviours are frequently performed in the context of reunion (although not exclusively [37]), and one proposed function of greetings is to update uncertain relationships between individuals following prolonged periods without social contact, and greetings are often observed in species that demonstrate high degree fission-fusion dynamics, such as male African elephants [22,34,36]. The tension and potential conflict of reunion (or meeting of completely novel individuals) during group fusion events is thought to be resolved by greeting behaviours, and individuals can communicate their intention to interact in an affiliative manner, as well as update previously insecure relationships (e.g., establish relative dominance status) via close contact assessments [36,38]. Greetings are also argued to serve other functions in other species, which may similarly apply to male African elephants. For example, greeting behaviours can be a mechanism to reconcile and diffuse conflicts [12,34,39], a means for individuals to communicate their awareness of dominance asymmetries (e.g., appeasement behaviours from subordinates to dominants [40]), and as a means of facilitating positive relationships, maintaining cooperation and reinforcing social bonds among members of groups [34,37,41].

This study explores trunk-to-SEO behaviours performed by male African elephants aggregating at hotspots of social activity along a river in an area spatially segregated from females. African elephants are a long-lived species, and males aggregate in mixed age groups [23,27]. Localised, but shareable resources in the environment such as these river hotspots provide aggregating males exposure to different ages of potential interactors, and hence opportunities for information exchange and social contact with a diverse set of new associates [42,43]. We were first interested in exploring how differences in age affect the rate at which elephants perform trunk-to-SEO behaviours, hypothesising that (i) males of different ages will perform trunk-to-SEO behaviours at divergent rates. Adolescents are in general more sociable than adult male African elephants; they are found in larger groups [28], and are less likely to travel alone [44]. If adolescents perform more trunk-to-SEO behaviours compared to adults, it may be that these behaviours are used to facilitate further social connections and interactions, or to obtain information about other males in the male social network to which adolescents are more recently dispersed and less stably established in [27,31]. Alternatively, if breeding age adult males perform more trunk-to-SEO behaviours than non-breeding age adolescents, it may be that the main function of these behaviours is for evaluation of sexual status of potential competitors, for example proximity to transmission to musth state [15]. Throughout this study, we also consider the organs targeted by the trunk separately from one another. The mouth, temporal gland, and genitals, may carry different olfactory information, and may therefore provide different information about a conspecific [11,15,16,17,18,19,45]. In addition, trunk-to-SEO behaviours can also be reciprocated [11], we therefore also explored whether age class influenced the likelihood of a subject’s trunk-to-SEO behaviour being a reciprocated event.

Secondly, we explored to what age of target trunk-to-SEO behaviours are directed, as an indicator of the potential function of these behaviours in male African elephant communication. We tested two alternative hypotheses. From adolescence through to adulthood, male elephants grow drastically [46], making relative dominance easy to establish between individuals separated by large age gaps. For males that are of similar age, we predicted that trunk-to-SEO behaviours may be important for clarifying less obvious dominance relationships [8], and we thus hypothesised that (ii) elephants will target age-matched elephants more with trunk-to-SEO behaviours than predicted by chance. Males may also prefer to associate with age-matched partners for cooperative benefits, such as access to size and strength matched sparring partners [23,28] and trunk-to-SEO behaviours between age-mates may also be used to facilitate new connections and communicate affiliative intent towards such partners. For our alternate hypothesis, we predicted that (iii) elephants will target the oldest males in the population with trunk-to-SEO behaviours more than predicted by chance. There is evidence that older male African elephants are the preferred nearest neighbours of males of all ages [28] and older males have a greater number of associates, and higher centrality in male social networks [23]. In fusion events of spotted hyenas, high ranking females are preferentially targeted with close contact greeting behaviours of the genitals, reflecting a preference for powerful allies and popular social contacts [34]. Similarly, as in older matriarchs in African elephant female groups, older, mature bulls may represent desirable contacts owing to potentially enhanced ecological and social experience and knowledge [23,44,47], and elephants may benefit from directing trunk-to-SEO behaviours to these high-value targets to initiate further contact, or obtain information on, for example, their individual identity or feeding behaviour [16,28]. Alternatively, male elephants may also preferentially direct trunk-to-SEO behaviours to older bulls in the case that this behaviour is primarily an appeasement gesture performed by subordinates to dominants to signal awareness of dominance asymmetry [10,12,40,48].

Finally, we compared how male African elephants direct trunk-to-SEO behaviours to targets based on their relative novelty to the actor. In societies with a high degree of fission-fusion dynamics such as male African elephants, these trunk behaviours could be used for reaffirming relationships following separations, or obtaining information about new, unknown individuals in a safe context [8,36]. Hence, we hypothesised that (iv) elephants will be more likely to direct trunk-to-SEO behaviours towards new individuals met at all-male aggregations at social hotspots, compared to elephants that they arrived at the hotspot in an all-male group with.

## 2. Methods

### 2.1. Data Collection

Subjects of the study were male African elephants aggregating at hotspots of male elephant social activity along the Boteti River, in Makgadikgadi Pans National Park (MPNP), Botswana, a recognised bull area with 98% male sightings [49]. Details regarding the nature (size, location, resources present) of these “social hotspots” can be found in [50].

Focal subjects were recorded for the duration of their stay within defined social hotspots using a video camcorder (JVC quad proof AVCHD). Video data were collected at staggered start times covering 08:00–18:30 between September 2015 and September 2018 (see [50] for further details of methods and sampling times). Male African elephants were categorized into 4 age classes: adolescents, 10–15 years and 16–20 years, and adults, 21–25 and 26+ years, based on shoulder height and overall body size [46], head size and shape, as well as tusk girth and extent of splay [51]. We randomly preselected the age class of the subject to be recorded for a particular follow, and the first elephant of the assigned age class to arrive at the hotspot since observers started the session, would be the subject of a focal animal sample. If more than 1 elephant arrived of the predetermined age class in the same group, the focal was selected at random from the choice of elephants. Video focal follow recordings of visits to the river were taken from individual elephants only once over the course of the study, with an individual’s identity determined using characteristics such as notches, tears, holes and venation in the ears, morphology of tusks, folds and wrinkles of the skin, and other body abnormalities.

A focal follow began either as the subject arrived over the bank slope, or as he entered the hotspot having moved from another stretch of river up or downstream of the hotspot. Focal follows were terminated when similar boundaries were crossed during departure. Focals could stay at social hotspots for several hours (average time spent at hotspot for focal elephants seen arriving and leaving via bank = 1 h 13 min, range = 9 min–7 h 5 min, SD = 59 min), over which time, the males present at aggregations with focals could be highly dynamic. Since individuals arriving in all-male groups tend to arrive within 10 min of one another [44], focal follows were subdivided into 10-min follows (e.g., a focal follow of an elephant staying 50 min at the hotspot, would produce five 10-min focal follows), to which a corresponding social context was assigned (see below), in order to capture the temporally dynamic nature of male aggregations at the hotspots. Only focal follows where the subject was exposed to at least 1 potential interactor during his stay at the river were used for the study.

Data collection methods allowed for good visibility of focal elephants [50]. However, if a subject was out of view from the camera for over 2 min of a 10-min focal animal follow, i.e., over 20% of time (*n* = 152), the focal follow was excluded from analyses. This gave a total sample size of 1223 10-min focal follows for analysis (10–15 years: 246, 16–20 years: 320, 21–25 years: 319, 26+ years: 338), from 240 individuals.

### 2.2. Scoring of Trunk to Scent-Emitting-Organ Behaviours

**Figure 1:**
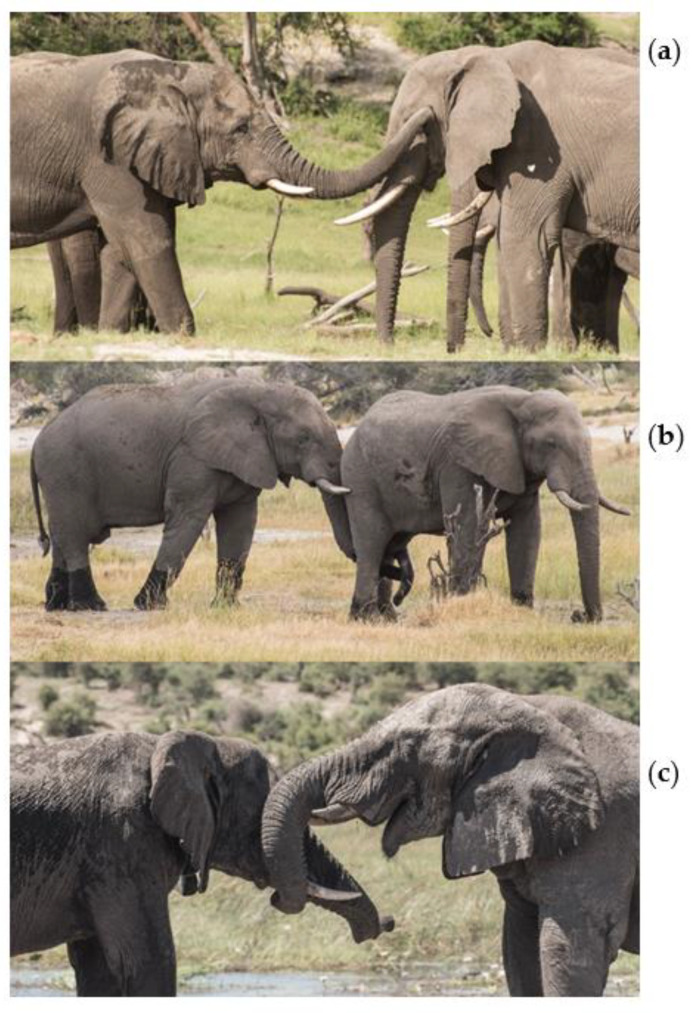
Example photos of close contact trunk-to-SEO behaviours, with trunk of a focal elephant directed to a target elephants (**a**) temporal gland, (**b**) genitals, and (**c**) mouth.

Video footage of focal follows was scored for behaviours by one researcher (CA) to standardise scoring. Focal follows were watched 3 times to verify behaviours. We recorded trunk behaviours performed by focal elephants that involved the direction of the trunk towards a target elephants’ temporal glands, genitals or mouth [11] (Figure 1).

We recorded trunk behaviours as events, recording the time each behaviour was performed, the target organ of the behaviour (Figure 1), the age class of the elephant targeted, and whether he was an elephant the focal arrived at the river with, or met new at the river (did not arrive in a group at the hotspot with). Trunk-to-SEO behaviours can be one-way, or reciprocated events between dyads [8,11]. Therefore, we also recorded whether a trunk-to-SEO behaviour directed by a focal to an individual was reciprocated or not (Supplementary Note S1 For determination of time window for reciprocating trunk behaviours). 

### 2.3. Social Context

The number of other elephants already present aggregating at the hotspot and their age classes were recorded at the time of a focal elephant’s arrival, as were the ages of those he arrived at the river with in a group. Furthermore, we continuously recorded elephants that arrived and left the hotspot by the riverbank or hotspot boundaries up or downstream during a focals stay, such that for every 10-min focal follow, there was a corresponding recording of the number of other elephants present at the hotspot with a subject as a potential interactor in that follow, and their age classes. 

No focal follows were collected from elephants identified to be in musth [14]. Furthermore, we excluded focal follows where a musth bull was present as a potential interactor at the hotspot (*n* = 11), due to the established consensus that males act differently in musth state, and non-musth males’ tendency to avoid musth males [14]. We also excluded a small number of follows where females were present as potential interactors (*n* = 11), since presence of females was a rare event in this bull area and was likely to influence choice of targets of and rates given of trunk-to-SEO behaviours.

### 2.4. Statistical Analysis

To determine how (i) rate of performing trunk-to-SEO behaviours varied with age class, Kruskal Wallis H tests were run, and where significant results were found, post hoc pairwise comparisons were made using Wilcoxon rank sum tests with corrections for multiple comparisons, to identify between which age classes these differences were driven. Rates of performing trunk-to-SEO behaviours were explored through number of behaviours made by subjects/hour, number of individuals targeted with behaviours/hour, number of individuals targeted with behaviours/potential interactor exposed to during a hotspot visit, and number of individuals targeted with behaviours/potential interactor/hour, as there are multiple ways to meaningfully measure the rate at which trunk-to-SEO behaviours are performed, which may need to be controlled for by number of potential interactors present, and sample duration. We similarly compared all these measures for the specific organ targeted separately, in case the age classes differed in their targeting of the different organs with trunk contacts. 

As a control, we also ran a generalised logistic mixed-effects model (GLMM) to explore whether number of other elephants present at hotspots with subjects predicted the likelihood of directing a trunk-to-SEO behaviour toward another animal during a 10-min focal follow. In this model “trunk-to-SEO behaviour performed in 10-min follow” (yes or no, dependent variable) was predicted by focal age class, the number of other elephants present at the male aggregation and the interaction term between the two (independent variables). Another GLMM, explored whether a focal elephant’s age class predicted whether a trunk-to-SEO behaviour was reciprocated by the target back to the focal. In this model, the independent variable was “trunk-to-SEO behaviour reciprocated” (yes or no), and independent variable was focal age class. Focal ID was included as a random effect in both these GLMMs.

We ran GLMMs to determine whether elephants directed trunk-to-SEO behaviours to particular age cohorts more than would be predicted by random assignment of these behaviours to elephants present in a subject’s social environment at the hotspot (all-male aggregations), with statistical significance determined using permutation-based null models. Only focal follows that had at least 1 individual from both categories of potential interactors present (see each hypothesis below), and had no more than 30 elephants present as potential interactors were included in models to assist with model convergence.

We first investigated if (ii) elephants directed trunk-to-SEO behaviours to age-matched elephants at hotspots more than predicted by random chance assignment of these behaviours to the elephants present with subjects at hotspots. We fit a GLMM with a binomial error structure and a logit link function, predicting “trunk-to-SEO behaviour given to individual” (dependent variable, yes or no) by whether a potential interactor (present in that 10-min focal follow) was age-matched or not to the focal elephant (independent variable). We ran four separate models: one including all trunk-to-SEO behaviours directed (3 target organs combined), as well as individually for the particular organ targeted (mouth, temporal gland, genitals). Binomial GLMMs were fit, and estimates obtained for the observed data set were compared to 10,000 randomised data sets. In these permutations, the age composition of the social environment was maintained (number of age-matched and non-age-matched present at the hotspot) in each 10-min follow, but trunk-to-SEO behaviours given were randomly shuffled between the individuals present in each permutation. We then ran the same models again, this time including focal age class as an interaction term, to investigate whether the age classes differed in their tendency to target age-matched males with trunk-to-SEO behaviours. In all models, focal ID was included as a random effect.

For our alternative hypothesis, we investigated whether (iii) elephants directed trunk-to-SEO behaviours to older, mature individuals (aged 26+ years) more than younger age classes of elephants. The structure of these GLMM’s, and methodology was identical to the above models concerning age-matched status. However, the independent variable was the age of the potential target of interaction (either aged 26+ years or not).

Finally, we explored whether (iv) elephants preferentially directed trunk-to-SEO behaviours to individuals that they did not arrive at the hotspot with compared to those with whom they did. These GLMMs predicted “trunk-to-SEO behaviour given to individual” (dependent variable, yes or no) by the “novelty” status of the elephants present during a focals stay at the river hotspot (arrived at the river in a travelling group with the focal, or was a new interactor met at the all-male aggregation). In these permutations, only elephants that were observed leaving and arriving via the riverbank (no arrival or departure from up or downstream of hotspot) were used for analysis. The “novelty” status of elephants was maintained in each permutation (number of elephants met new versus arrived with exposed to), but trunk-to-SEO behaviours were randomly shuffled amongst the individuals present in each permutation. Binomial GLMMs were fit, and estimates obtained for the observed data set were compared to 10,000 randomised data sets. Again, models were also run for the target organs separately, and rerun to include age class of the subject elephant as an interaction term. Focal ID was included as a random effect in all models.

## 3. Results

Considering all trunk-to-SEO behaviours together, males of divergent age classes performed trunk behaviours at different rates (Supplementary Table S1 for Kruskal–Wallis H test results, Supplementary Table S2 for means and standard deviations of trunk-to-SEO behaviours performed by the different age classes). This was driven in all cases by the two adolescent age classes performing trunk-to-SEO behaviours of conspecifics at significantly higher rates than the two adult age classes (Supplementary Tables S3–S6 for significant pairwise comparisons using Wilcoxon rank sum tests). 

**Figure 2:**
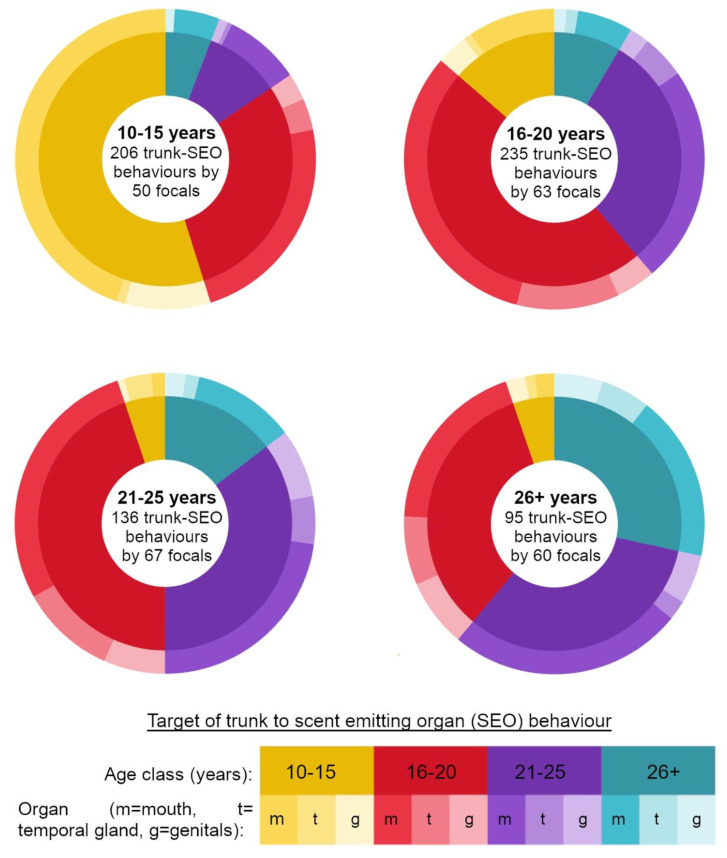
Donut charts summarising all observed trunk-to-SEO behaviours directed by focal elephants of different ages. Inner rings indicate age class targeted for behaviour, and outer rings the target organ.

Trunk-to-SEO behaviours were largely dominated by trunk-to-mouth behaviours (Figure 2). Considering only trunk-to-mouth behaviours, males of divergent age classes performed this behaviour at different rates (Supplementary Table S1 for Kruskal–Wallis H test results, Supplementary Table S7 for means and standard deviations of trunk-to-mouth behaviours performed by the different age classes). This was again driven in all cases by males from both the adolescent age classes performing trunk-to-mouth behaviours at significantly higher rates than males from both adult age classes (Supplementary Tables S8–S11 for significant pairwise comparisons using Wilcoxon rank sum tests). Age class had no effect on the rates at which elephants directed the trunk to conspecifics’ temporal glands or genitals (Supplementary Table S1 for Kruskal–Wallis H test results, Supplementary Tables S12 and S13 for means and standard deviations of trunk-to-temporal-gland and genital behaviours performed by the different age classes respectively).

For all age classes, the likelihood that a trunk-to-SEO behaviour was performed in a 10-min follow was unaffected by the total number of elephants present with the subject at all-male aggregations at hotspots (Supplementary Figure S1).

Age class of the subject significantly predicted whether a trunk-to-SEO behaviour was reciprocated. Higher probabilities of reciprocation were observed in adult age classes, with 10–15-year-olds having significantly lower probabilities of these trunk behaviours being reciprocated events than all other age classes (Probability of trunk-to-SEO behaviour being a reciprocated event: 10–15 years: 0.298; 16–20 years: 0.432; 21–25 years: 0.568; 26+ years: 0.537; Supplementary Table S14 for GLMM output for significant differences between age classes).

### 3.1. Elephants Preferentially Targeted Age-Matched Individuals with Trunk to Scent-Emitting-Organ Behaviours at Social Hotspots

Whether a potential interactor was age-matched to the subject elephant predicted the likelihood of the subject directing a trunk-to-SEO behaviour to him (permutation-based likelihood ratio test of GLMM, χ^2^ (1) = 5.485^−12^ , *p* < 0.001), with elephants directing these behaviours to age-matched individuals more than predicted by chance (Supplementary Table S15). Considering the target organs independently, whether a potential interactor was age-matched to the subject elephant predicted the likelihood of the subject directing a trunk-to-mouth and trunk-to-genital behaviour to him (permutation-based likelihood ratio test of GLMM, trunk-to-mouth: χ^2^ (1) = 4.803^−10^  , *p* < 0.001 , trunk-to-genitals χ^2^ (1) = 0.004, *p* = 0.005), but not a trunk-to-temporal-gland behaviour (permutation-based likelihood ratio test of GLMM: χ^2^ (1) = 0.134 , *p* = 0.198), with elephants preferably targeting age-matched elephants with trunk-to-mouth and trunk-to-genital behaviours (Supplementary Table S15).

**Figure 3:**
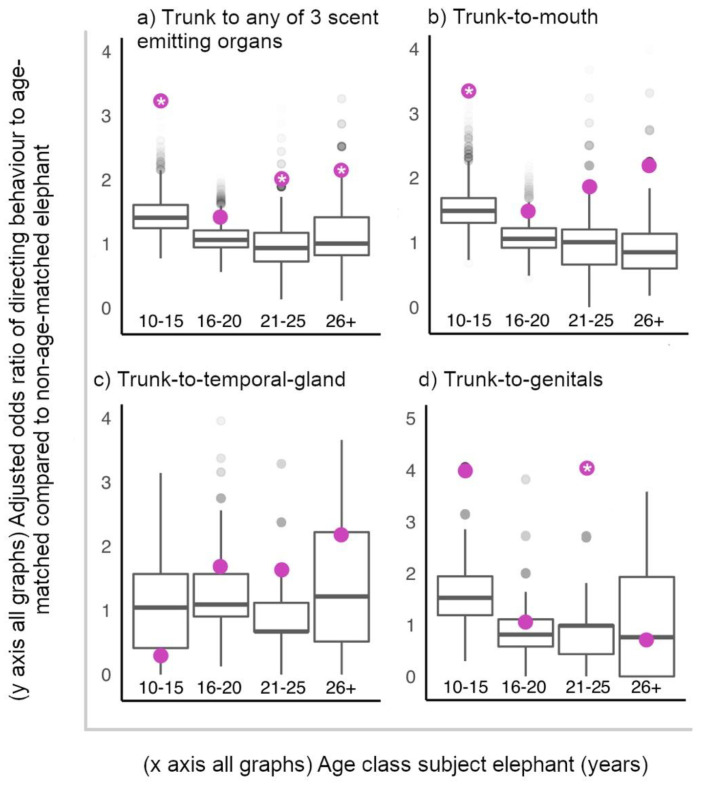
Observed adjusted odds ratios of elephants directing a trunk-to-SEO behaviour to an age-matched elephant relative to non-age-matched elephant (purple circles), plotted against random permuted adjusted odds ratios of directing a trunk-to-SEO behaviour to an age-matched relative to non-age-matched elephant (boxplots with median, interquartile range, minimum and maximum values). Significant permutation-based adjusted odds ratios indicated with “*”. (**a**) Considering all target organs together, all ages were more likely to target age-matched elephants relative to non-age-matched elephants with trunk-to-SEO behaviours than expected by chance, except older adolescents (16–20 years), who targeted age-matched elephants as expected by random chance (permutation-based observed adjusted odds ratio of targeting age-matched relative to non-age-matched elephant with trunk-to-SEO behaviour: 10–15 years = 3.268, *p* < 0.001; 16–20 years = 1.454, *p* = 0.085; 21–25 years = 2.056, *p* = 0.014; 26+ years = 2.185, *p* = 0.048 (Supplementary Table S16 for randomised 95% confidence intervals)). (**c**) All age classes directed trunk-to-temporal-gland behaviours to age-matched elephants as expected by random chance, (**b**) 10–15-year-olds directed trunk-to-mouth behaviours to age-matched elephants more than expected by random chance, and (**d**) 21–25-year-olds directed trunk-to-genitals behaviours to age-matched males more than expected by random chance (Supplementary Table S16 for observed adjusted odds ratios, 95% confidence intervals and *p* values for each age class and target organ).

All ages targeted age-matched individuals with trunk-to-SEO behaviours more than predicted by random assignment of these behaviours to individuals present, apart from 16–20-year-olds, who targeted age-matched individuals with trunk-to-SEO behaviours as expected by random chance (Figure 3). Considering the target organs independently, 10–15-year-olds directed trunk-to-mouth behaviours to age-matched elephants, and 21–25-year-olds directed trunk-to-genitals of age-matched males more than expected by random chance (Figure 3).

### 3.2. Elephants Did Not Preferentially Target Older Individuals with Trunk to Scent-Emitting-Organ Behaviours at Social Hotspots

Whether an elephant target was aged 26+ or not did not predict the likelihood of a subject elephant directing a trunk-to-SEO behaviour to him (permutation-based likelihood ratio test of GLMM, χ^2^ (1) = 0.212, *p* = 0.199). Considering the organs targeted separately, whether an elephant was aged 26+ or not did not predict the likelihood of the subject directing his trunk to his genitals (permutation-based likelihood ratio test of GLMM: χ^2^ (1) = 0.295, *p* = 0.336), temporal gland (χ^2^ (1) = 0.949, *p* = 0.960), or mouth (χ^2^ (1) = 0.225, *p* = 0.214). Elephants directed all trunk-to-SEO behaviours to elephants aged 26+ years as predicted by random assignment of these behaviours to elephants present at all-male aggregations in the social hotspot environment (Supplementary Table S17).

**Figure 4:**
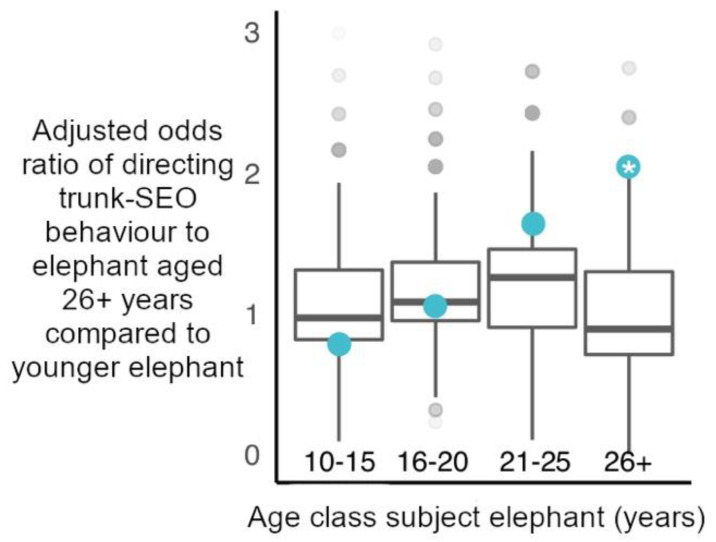
Observed adjusted odds ratios of elephants directing a trunk-to-SEO behaviour to a mature bull (26+ years) relative to younger elephant (blue circles), plotted against random permuted adjusted odds ratios of directing a trunk-to-SEO behaviour to a mature bull relative to younger elephant (boxplots with median, interquartile range, minimum and maximum values). Only elephants aged 26+ years directed these behaviours to fellow mature bulls more than expected by chance (Significant permutation-based adjusted odds ratio (*): 2.077, *p* = 0.042 (Supplementary Table S18 for observed adjusted odds ratios, 95% confidence intervals and *p* values for all ages)).

Concerning individual age classes, in line with age-matched models, elephants aged 26+ years directed trunk-to-SEO behaviours to fellow elephants aged 26+ years more than predicted by chance (Figure 4). All other age classes targeted elephants aged 26+ years with trunk-to-SEO behaviours within the range predicted by random chance (Figure 4). Considering the target organs separately, all age classes directed trunk behaviours to the mouth, temporal glands and mouth of elephants aged 26+ years as expected by random chance (Supplementary Figure S2 and Table S18).

### 3.3. Elephants Did Not Preferentially Target New Individuals with Trunk to Scent-Emitting-Organ Behaviours at Social Hotspots

**Figure 5:**
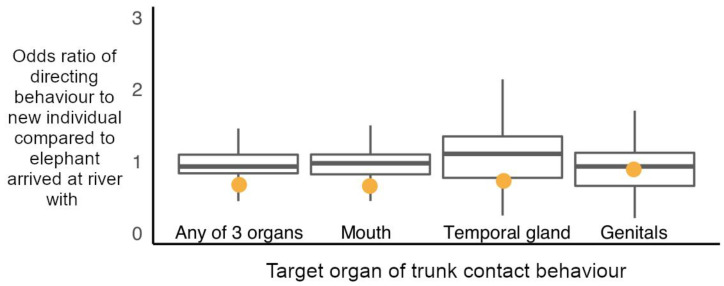
Observed odds ratio of directing trunk-to-SEO behaviours to new individuals encountered at the river compared to individuals the subject arrived with during a social hotspot visit (orange circles), plotted against the randomly permuted odds ratios of directing a trunk-to-SEO behaviour to elephants met new compared to arrived with at social hotspot (boxplots with median, interquartile range, minimum and maximum values). Elephants directed trunk-to-SEO behaviours to new elephants as predicted by random assignment of behaviours to elephants present during a social hotspot visit (permutation-based observed odds ratios: trunk to any of 3 organs (trunk-to-SEO) = 0.711, *p* = 0.182; trunk-to-mouth = 0.693, *p* = 0.145; trunk-to-temporal-gland = 0.770, *p* = 0.521; trunk-to-genitals = 0.929, *p* = 0.947 (Supplementary Table S20 for randomised 95% confidence intervals)).

There was no change in an elephant’s probability of directing a trunk-to-SEO behaviour over the time course of his stay at a hotspot, nor any evidence that elephants directed these behaviours more upon their initial arrival at hotspots (Supplementary Table S19, Supplementary Figure S3). Furthermore, whether an elephant arrived at the river in a group with the subject elephant, or whether the subject was exposed to an elephant as a new potential interactor at the river did not predict the likelihood of the subject directing a trunk-to-SEO behaviour to him (permutation-based likelihood ratio test of GLMM, χ^2^ (1) = 0.135, *p* = 0.107; Figure 5). The observed odds of targeting new individuals with trunk-to-SEO behaviours compared to elephants arrived with at the river fell within the range predicted by random assignment of behaviours to elephants present for all age classes of elephants (Supplementary Figure S4).

Considering the organs targeted with trunk-to-SEO behaviours individually, whether an elephant arrived at the river in a group with the subject elephant, or whether the subject was exposed to an elephant as a new potential interactor at the river did not predict the likelihood of the subject directing the trunk to a target’s mouth (permutation-based likelihood ratio test of GLMM, χ^2^ (1) = 0.120, *p* = 0.116; Figure 5), temporal glands (permutation-based likelihood ratio test of GLMM, χ^2^ (1) = 0.535, *p* = 0.525, Figure 5) or genitals (permutation-based likelihood ratio test of GLMM, χ^2^ (1) = 0.868, *p* = 0.843, Figure 5).

## 4. Discussion

Male African elephants from adolescent age classes directed close contact trunk-to-SEO behaviours to conspecifics at higher rates than elephants from adult age classes. As adolescents are more likely to be recently dispersed from their natal herd [27], they may have a greater need to obtain information about other males in the male social network, including identities of individuals [52], and relative dominance rank, compared to adults that are more established and stable in the network [31]. Similarly, adolescent male African elephants are more sociable in general than adults [28], and may perform more of these trunk behaviours to assist in establishing new contacts and initiating further affiliative interactions with social companions, a pattern also seen in other species that perform greeting behaviours [38]. Adolescent male African elephants also assess urine cues of conspecifics in the environment at greater rates than adults [53]. As part of the intense learning that is undergone in adolescence, adolescents may need to map phenotype features as well as individual identities (there is evidence elephants hold long term memory of individuals by their unique chemical signatures [18,54,55]) of particular same-sex conspecifics to their corresponding chemosignals via close contact olfactory assessments (trunk-to-SEO behaviours). In other words, as part of the recognition process, adolescents may need to learn which olfactory features belong to which individuals and phenotypes when forming mental templates, whilst adults may have already learnt this [56,57].

However, of the organs targeted, adolescents only directed trunk-to-SEO behaviours to the mouth at greater rates than adults. Trunk-to-genitals and trunk-to-temporal-gland behaviours appeared to be equally important to elephants of all ages. Elephant calves and juveniles commonly place their trunks in the mouth of their mothers and other females in their natal families as a conciliatory gesture, or to solicit, sample or steal food items [12,16,17]. Among females in female groups, trunk-to-mouth is the most common component of affiliative interactions [12], and adolescent males may continue to perform this familial behaviour post dispersal, with the rate declining with age as the male adopts more adult male behaviour. Since only trunk-to-mouth behaviours were influenced by the age class of our subjects, it is possible that trunk-to-genitals and trunk-to-temporal-gland behaviours may communicate information on different aspects of phenotype, or as tactile and gestural signals may be used to communicate divergent social messages to that of trunk-to-mouth behaviours. Similarly, in rats, facial sniffing is thought to be an appeasement signal, but not genital or flank sniffing [10].

Our results suggest that at least in African elephant bull areas segregated from females, trunk-to-SEO behaviours are not primarily used for monitoring the sexual status of potential competitors, since none of the target organs were contacted at greater rates by breeding age adult males (adolescents are unlikely to be of a competitive age for mating [29,30]). Musth (and hence sexual) status, as well as proximity to transitioning to musth state, is believed to be signaled in the urine, temporal glands and breath, i.e., all the target organs [19,45,58,59], although receivers’ ability to detect musth has only been confirmed in urine [15]. Although adolescent males can show a particular interest in musth males, watching and following them, perhaps for learning of sex-specific behaviours (Personal Communication, Reviewer 2), our later findings concerning mature males not being preferentially targeted with trunk behaviours to any organs suggests the individuals most likely to be transitioning to a sexual, musth state were not those preferentially targeted with trunk behaviours, providing further support for our argument that sexual assessment is not the prime motive of these behaviours.

Previous research found that the decline in olfactory investigation of urine cues in the environment from adolescence to adulthood in male African elephants is compensated for by adults only investigating relevant cues [53]. This was also supported in our study-most notably by the very low numbers of trunk-to-SEO behaviours being made of 10–15-year-olds by adult males. These young adolescents are likely to represent both a low threat to adult males, as well as non-valuable social companions concerning learning opportunities or sparring partners. Similarly, the fact that young adolescents, 10–15 were less likely than all other ages to have their trunk-to-SEO behaviours reciprocated also suggests their low value as social contacts/sources of information to age classes older than their own.

Alternatively, because with increasing age males have greater distances to their nearest neighbours on average [28], adults may perform less trunk-to-SEO behaviours simply because adult males had fewer opportunities to perform such close contact behaviours. Similarly, adult males may be less likely to engage in the intimate behaviours that provide opportunity for these trunk directed contacts. For example, trunk-to-mouth and trunk-to-temporal-gland behaviours were sometimes performed during sparring bouts, an activity engaged in more by adolescents than adults [28]. However, the importance of these features seems small considering only trunk-to-mouth behaviours were affected by age. If lack of opportunities for close contact behaviours explained our results, we would also expect an influence of age class on trunk behaviours directed to the temporal glands and genitals, which we did not find.

In the current study, it was observed that when an elephant received a trunk-to-genital behaviour, he sometimes ceased movement and “allowed” himself to be assessed in the case where he was younger than the director of the behaviour. In contrast, where the targeted individual was an adult, and older or age-matched to the subject, trunk-to-genital behaviours were sometimes responded to with kick-backs and tail swatting behaviour to the subject, suggesting dominance related interactions occur alongside this behaviour. Trunk contacts directed to the mouth and temporal gland did not appear to trigger any dominance related behaviours between males and appeared to have a far more benign and mutual reception, and were performed in a variety of contexts from during drinking and feeding on riverbed substrate, to during sparring bouts. In a recently formulated ethogram of elephant behaviour and communication, a large emphasis was drawn to the multiple contexts in which trunk-to-SEO behaviours are performed [12]. We suggest future studies consider the behaviours of elephants in the interaction immediately prior to and after the trunk-to-SEO behaviour is performed, as signals can vary in meaning depending on the context in which they are performed [60].

All ages of elephants preferentially directed trunk-to-SEO behaviours to age-matched males at male-aggregations, except older adolescents, aged 16–20 years. If considering these tactile and olfactory behaviours as greeting behaviours between males, a suggested function may be that these behaviours are used to initiate and facilitate further association and interaction with partners, e.g., for sparring activity, a behaviour important for testing and developing skills for competitive fighting [61]. Males in many species, including elephants, prefer to spar with age-matched individuals [23,62]. Alternatively, these greeting type behaviours may also be used to assess individuals similar in dominance rank in a safe and ritualised context. Trunk-to-SEO behaviours may assist in discerning relative dominance through assessment of olfactory cues (for example levels of hormones and other volatile compounds that reflect social dominance, such as androgens like testosterone [3,19,63]) in otherwise similarly sized and strength matched individuals. Male hamadryas baboons *Papio hamadryas* also use greetings as an assessment strategy, with rivals matched in dominance and competitive abilities exchanging more greetings than those un-matched in competitive ability [64], and male mantled howlers *Alouatta palliata* are more likely to greet conspecific males close in dominance rank to themselves [8].

16–20-year-olds targeted age-matched males with trunk-to-SEO behaviours within the range predicted by random chance. In addition, 16–20 had the lowest odds of directing these behaviours to age-matched compared to non-age-matched males out of all the 4 age classes. This may represent a widening of interests concerning beneficial social partners in late adolescence, and a period where African elephants are not so focused on peer specific relationships, and are less selective of who they target for obtaining information on or initiating interaction with. This compliments findings of Chiyo et al. [23], that whilst adult age classes of African elephants associated with age-mates preferentially, adolescents associated with their own age class at random. Alternatively, this result may reflect the fact that age was recorded categorically rather than continuously. Elephants at the older end of 16–20 years may be closer in age (hence better “age-matched”) to the youngest individuals in the category 21–25 years than to some members of their own age class, and vice versa, elephants at the younger end of 16–20 years may be closer in age to the oldest individuals in the 10–15 years category than to some members of their own age class. In this way, elephants within the 16–20-year-old class may have experienced a diffusing of the effect of targeting age-mates with trunk-to-SEO behaviours. At least that is in comparison to the youngest (10–15 years) and oldest (26+ years) age classes, who are only affected on one end of their category by the possibility that a similar aged interactor is in fact categorised into an adjacent age category. However, by this logic, elephants aged 21–25 years would also direct trunk-to-SEO behaviours to age-matched males as random chance, which we did not find. Regardless, future study may wish to consider age as a continuous variable, with age-match status measured continuously as absolute age difference, to avoid such uncertainty with interpreting results.

The oldest African elephants in all-male aggregations at social hotspots were not preferentially targeted with trunk-to-SEO behaviours by younger males. This suggests male elephants are not primarily using these trunk behaviours to initiate contact with or obtain information such as identity and feeding history from older, more experienced individuals that may be high value social partners. Furthermore, these behaviours do not appear to be appeasement gestures directed to dominants [48]. Despite all ages of male African elephants demonstrating a preference to maintain close proximity to older males [28], we found no evidence that elephants preferentially targeted them with trunk-to-SEO behaviours, suggesting males learn from older, experienced males via other modalities and not close contact trunk-to-SEO behaviours. We recommend exploring whether more “eavesdropping” type methods are used for learning from older males [65]. Visual cues (observing older males), and auditory cues (listening to older males) may be more likely to be important modes of inter-generational social learning in male African elephants [66,67].

Finally, contrary to our hypothesis, males did not preferentially target new social companions met at the hotspot compared to those they had been seen to have been associating with, having arrived in a group together to the hotspot with trunk-to-SEO behaviours. Trunk-to-SEO behaviours thus do not seem to be primarily used by male African elephants as a way to peacefully initiate contact between, or obtain information on, unknown individuals, or individuals that have been separated for prolonged periods of time in this fission-fusion society [8,36]. Additionally, over the time period of an elephant’s stay at the river, there were no changes in the probability of performing these behaviours, suggesting trunk-to-SEO behaviours are general, continuous olfactory assessments and/or tactile contacts between individuals sharing the same ecological space. This is in stark contrast to the vivid and high-energy reunion events that can occur at fusion events of females in African elephant family groups [12]. If viewed as a greeting behaviour, the performance of these trunk-to-SEO behaviours better matches the putative function for spatially tolerant gregarious males of testing willingness to interact, facilitating positive relationships, and assessing aspects of phenotype of same-sex conspecifics [37,68].

## 5. Conclusions

This paper explored the potential functions of close contact trunk-to-SEO behaviours between male African elephants of divergent age and familiarity to one another, living in a fission-fusion society. In this male-dominated area, it is unlikely males primarily use trunk-to-SEO behaviours to assess reproductive condition of competitors. Trunk-to-mouth behaviours were performed more by adolescent than adult subjects, and likely communicate different information between signaler and receiver than trunk-to-genitals and trunk-to-temporal-gland behaviours. Male elephants of all ages, apart from older adolescents, preferentially targeted age-mates for trunk-to-SEO behaviours, and we found no evidence that males directed trunk-to-SEO behaviours preferentially to novel, unfamiliar individuals met at social hotspots. Our results suggest male elephants may use trunk-to-SEO behaviours to facilitate further positive interaction with other males or assess aspects of phenotype (such as relative dominance) between males generally occupying the same ecological space, rather than as a benign “first contact” signal directed at novel social partners.

## Data Availability

Due to the sensitive nature of reporting on elephant locations and numbers, the data that support the findings of this study are available on reasonable request from the corresponding author.

## Supplementary Materials

The following are available online at https://www.mdpi.com/article/10.3390/ani11092718/s1, Note S1: Definition of reciprocated trunk-SEO behaviours, Table S1: Output of Kruskal-Wallis H tests for each method of measuring rate of performing of trunk-to-SEO behaviours by age class of focal, Table S2: Table of means and standard deviations of trunk-to-SEO behaviours performed by focals of different age classes during a visit to social hotspot, Table S3: Post hoc pairwise comparisons using Wilcoxon rank sum test with continuity correction, showing differences between the 4 age classes concerning trunk-to-SEO behaviours performed per hour, Table S4: Post hoc pairwise comparisons using Wilcoxon rank sum test with continuity correction, showing differences between the 4 age classes concerning number of individuals targeted with trunk-to-SEO behaviours per hour, Table S5: Post hoc pairwise comparisons using Wilcoxon rank sum test with continuity correction, showing differences between the 4 age classes concerning number of individuals targeted with trunk-to-SEO behaviours per potential interactor exposed to during visit to social hotspot, Table S6: Post hoc pairwise comparisons using Wilcoxon rank sum test with continuity correction, showing differences between the 4 age classes concerning number of individuals targeted with trunk-to-SEO behaviours per potential interactor per hour, Table S7: Table of means and standard deviations of trunk-to-mouth behaviours performed by focals of different age classes during a visit to social hotspot, Table S8: Pairwise comparisons using Wilcoxon rank sum test with continuity correction, showing differences between the 4 age classes concerning trunk-to-mouth behaviours performed per hour, Table S9: Post hoc pairwise comparisons using Wilcoxon rank sum test with continuity correction, showing differences between the 4 age classes concerning number of individuals targeted with trunk-to-mouth behaviours per hour, Table S10: Post hoc pairwise comparisons using Wilcoxon rank sum test with continuity correction, showing differences between the 4 age classes concerning number of individuals targeted with trunk-to-mouth behaviours per potential interactor exposed to during visit to social hotspot, Table S11: Post hoc pairwise comparisons using Wilcoxon rank sum test with continuity correction, showing differences between the 4 age classes concerning number of individuals targeted with trunk-to-mouth behaviours per potential interactor per hour, Table S12: Table of means and standard deviations of trunk-to-temporal-gland behaviours performed by focals of different age classes during a visit to social hotspot, Table S13: Table of means and standard deviations of trunk-to-genitals behaviours performed by focals of different age classes during a visit to social hotspot, Table S14: GLMM output of likelihood of a trunk-to-SEO behaviour being a reciprocated event, predicted by age class of the focal subject, Table S15: Observed odds ratios and permutation based significances of elephants targeting an age-matched individual relative to non-age-matched individual with trunk-to-SEO behaviours of different target organs, Table S16: Observed adjusted odds ratios and permutation based significances of elephants of different age class targeting an age-matched relative to non-age-matched individual with trunk-to-SEO behaviours of different target organs, Table S17: Observed odds ratios and permutation based significances of elephants targeting an elephant aged 26+ years relative to a younger male with trunk-to-SEO behaviours of different target organs, Table S18: Observed adjusted odds ratios and permutation based significances of elephants of different age classes targeting a male aged 26+ years relative to younger male with trunk-to-SEO behaviours of different target organs, Table S19: GLMM outputs of likelihood of elephant directing trunk-to-SEO behaviours to conspecific during a 10-min focal follow by different time conditions during his visit to social hotspot, Table S20: Observed odds ratios and permutation-based significances of elephants targeting an elephant met new at the river relative to elephant arrived at river with, with trunk-to-SEO behaviours of different target organs, Figure S1: The number of other elephants present with the focal did not significantly effect probability of directing a trunk-to-SEO behaviour in a 10-min follow, Figure S2: Observed adjusted odds ratios of elephants directing trunk-to-SEO behaviours to an elephant aged 26+ years relative to a younger elephant (blue circles), plotted against randomly permuted adjusted odds ratios of directing trunk-to-SEO behaviour to elephant aged 26+ years relative to a younger elephant (boxplots with median, interquartile range, minimum and maximum values), Figure S3: There was no change in a focals probability of directing a trunk-to-SEO behaviour to a conspecific over the course of his stay at a focal hotspot.

## References

[1] Eisenberg J.F., Kleiman D.G. (1972). Olfactory Communication in Mammals. Annu. Rev. Ecol. Syst..

[2] Kelliher K.R. (2007). The Combined Role of the Main Olfactory and Vomeronasal Systems in Social Communication in Mammals. Horm. Behav..

[3] Gosling L.M., Roberts S.C. (2001). Scent-marking by male mammals: Cheat-proof signals to competitors and mates. Advances in the Study of Behavior.

[4] Gazit I., Terkel J., Zuri I. (1997). Effect of Scent-Marking in Delaying Territorial Invasion in the Blind Mole-Rat Spalax Ehrenbergi. Behaviour.

[5] Ferkin M.H., Lee D.N., Leonard S.T. (2004). The Reproductive State of Female Voles Affects Their Scent Marking Behavior and the Responses of Male Conspecifics to Such Marks. Ethology.

[6] East M.L., Hofer H., Wickler W. (1993). The Erect Penis Is a Flag of Submission in a Female-Dominated Society: Greetings in Serengeti Spotted Hyenas. Behav. Ecol. Sociobiol..

[7] van den Bos R., de Cock Buning T. (2010). Social Behaviour of Domestic Cats (*Felis lybica* f. *Catus* L.): A Study of Dominance in a Group of Female Laboratory Cats. Ethology.

[8] Dias P.A.D., Rodríguez Luna E., Canales Espinosa D. (2008). The Functions of the “Greeting Ceremony” among Male Mantled Howlers (Alouatta Palliata) on Agaltepec Island, Mexico. Am. J. Primatol..

[9] Baan C., Bergmüller R., Smith D.W., Molnar B. (2014). Conflict Management in Free-Ranging Wolves, Canis Lupus. Anim. Behav..

[10] Wesson D.W. (2013). Sniffing Behavior Communicates Social Hierarchy. Curr. Biol..

[11] Poole J.H., Granli P. (2011). Signals, Gestures, and Behavior of African Elephants. The Amboseli Elephants.

[12] Poole J.H., Granli P. (2021). The Elephant Ethogram. https://www.elephantvoices.org/elephant-ethogram/search-portal/options.html.

[13] Schulte B.A., Bagley K., Correll M., Gray A., Heineman S.M., Loizi H., Malament M., Scott N.L., Slade B.E., Stanley L., Mason R.T., LeMaster M.P., Müller-Schwarze D. (2005). Assessing chemical communication in elephants. Chemical Signals in Vertebrates 10.

[14] Poole J.H. (1987). Rutting Behavior in African Elephants: The Phenomenon of Musth. Behaviour.

[15] Hollister-Smith J.A., Alberts S.C., Rasmussen L.E.L. (2008). Do Male African Elephants, Loxodonta Africana, Signal Musth via Urine Dribbling?. Anim. Behav..

[16] Lee P.C., Moss C.J. (1999). The social context for learning and behavioural development among wild African elephants. Mammalian Social Learning.

[17] Lee P.C., Moss C.J. (2011). “Calf Development and Maternal Rearing Strategies”. In The Amboseli Elephants: A Long-Term Perspective on a Long-Lived Mammal.

[18] Bates L.A., Sayialel K.N., Njiraini N.W., Poole J.H., Moss C.J., Byrne R.W. (2008). African Elephants Have Expectations about the Locations of Out-of-Sight Family Members. Biol. Lett..

[19] Rasmussen L.E.L., Wittemyer G. (2002). Chemosignalling of Musth by Individual Wild African Elephants (*Loxodonta Africana*): Implications for Conservation and Management. Proc. R. Soc. Lond. B.

[20] Andersson M., Iwasa Y. (1996). Sexual Selection. Trends Ecol. Evol..

[21] Wobeser G.A. (2005). Essentials of Disease in Wild Animals.

[22] Aureli F., Schaffner C.M., Boesch C., Bearder S.K., Call J., Chapman C.A., Connor R., Fiore A.D., Dunbar R.I.M., Henzi S.P. (2008). Fission-Fusion Dynamics: New Research Frameworks. Curr. Anthropol..

[23] Chiyo P.I., Archie E.A., Hollister-Smith J.A., Lee P.C., Poole J.H., Moss C.J., Alberts S.C. (2011). Association Patterns of African Elephants in All-Male Groups: The Role of Age and Genetic Relatedness. Anim. Behav..

[24] Wittemyer G., Douglas-Hamilton I., Getz W.M. (2005). The Socioecology of Elephants: Analysis of the Processes Creating Multitiered Social Structures. Anim. Behav..

[25] Archie E.A., Moss C.J., Alberts S.C. (2006). The Ties That Bind: Genetic Relatedness Predicts the Fission and Fusion of Social Groups in Wild African Elephants. Proc. R. Soc. B.

[26] Goldenberg S.Z., de Silva S., Rasmussen H.B., Douglas-Hamilton I., Wittemyer G. (2014). Controlling for Behavioural State Reveals Social Dynamics among Male African Elephants, Loxodonta Africana. Anim. Behav..

[27] Lee P.C., Poole J.H., Njiraini N., Sayialel C.N., Moss C.J. (2011). Male social dynamics. The Amboseli Elephants: A Long-Term Perspective on a Long-Lived Mammal.

[28] Evans K.E., Harris S. (2008). Adolescence in Male African Elephants, Loxodonta Africana, and the Importance of Sociality. Anim. Behav..

[29] Hollister-Smith J.A., Poole J.H., Archie E.A., Vance E.A., Georgiadis N.J., Moss C.J., Alberts S.C. (2007). Age, Musth and Paternity Success in Wild Male African Elephants, Loxodonta Africana. Anim. Behav..

[30] Poole J.H., Lee P.C., Njiraini N., Moss C.J. (2011). Longevity, competition and musth: A long-term perspective on male reproductive strategies. The Amboseli Elephants: A Long-Term Perspective on a Long-Lived Mammal.

[31] Murphy D., Mumby H.S., Henley M.D. (2020). Age Differences in the Temporal Stability of a Male African Elephant (Loxodonta Africana) Social Network. Behav. Ecol..

[32] O’Connell-Rodwell C.E., Wood J.D., Kinzley C., Rodwell T.C., Alarcon C., Wasser S.K., Sapolsky R. (2011). Male African Elephants (Loxodonta Africana) Queue When the Stakes Are High. Ethol. Ecol. Evol..

[33] Harrington F.H., Asa C.S. (2013). Wolf communication. Wolves: Behavior, Ecology, and Conservation.

[34] Smith J.E., Powning K.S., Dawes S.E., Estrada J.R., Hopper A.L., Piotrowski S.L., Holekamp K.E. (2011). Greetings Promote Cooperation and Reinforce Social Bonds among Spotted Hyaenas. Anim. Behav..

[35] Whitham J.C., Maestripieri D. (2003). Primate Rituals: The Function of Greetings between Male Guinea Baboons: Greetings between Male Baboons. Ethology.

[36] Aureli F., Schaffner C.M. (2007). Aggression and Conflict Management at Fusion in Spider Monkeys. Biol. Lett..

[37] Dal Pesco F., Fischer J. (2018). Greetings in Male Guinea Baboons and the Function of Rituals in Complex Social Groups. J. Hum. Evol..

[38] Colmenares F., Hofer H., East M.L. (2000). Greeting ceremonies in baboons and hyenas. Natural Conflict Resolution.

[39] Aureli F., Cords M., van Schaik C.P. (2002). Conflict Resolution Following Aggression in Gregarious Animals: A Predictive Framework. Anim. Behav..

[40] Laporte M.N.C., Zuberbühler K. (2010). Vocal Greeting Behaviour in Wild Chimpanzee Females. Anim. Behav..

[41] Rütten S., Fleissner G. (2004). On the Function of the Greeting Ceremony in Social Canids—Exemplified by African Wild Dogs Lycaon Pictus. IUCN/SSC Canid Specialist Group. http://www.canids.org/canidnews/7/Greeting_ceremony_in_canids.pdf.

[42] Fishlock V., Lee P.C. (2013). Forest Elephants: Fission–Fusion and Social Arenas. Anim. Behav..

[43] Fishlock V., Caldwell C., Lee P.C. (2016). Elephant Resource-Use Traditions. Anim. Cogn..

[44] Allen C.R.B., Brent L.J.N., Motsentwa T., Weiss M.N., Croft D.P. (2020). Importance of Old Bulls: Leaders and Followers in Collective Movements of All-Male Groups in African Savannah Elephants (Loxodonta Africana). Sci. Rep..

[45] Rasmussen L.E.L., Riddle H.S. (2004). Elephant breath: Clues about health, disease, metabolism and social signals. J. Eleph. Manag. Assoc..

[46] Lee P.C., Moss C.J. (1995). Statural Growth in Known-Age African Elephants (Loxodonta Africana). J. Zool..

[47] McComb K., Shannon G., Durant S.M., Sayialel K., Slotow R., Poole J., Moss C. (2011). Leadership in Elephants: The Adaptive Value of Age. Proc. R. Soc. B.

[48] Preuschoft S. (1999). Are Primates Behaviorists: Formal Dominance, Cognition, and Free-Floating Rationales. J. Comp. Psychol..

[49] Evans K.E. (2019). Elephants for Africa: Male Savannah Elephant Loxodonta Africana Sociality, the Makgadikgadi and Resource Competition. Int. Zoo Yb..

[50] Allen C.R.B., Croft D.P., Brent L.J.N. Reduced Older Male Presence Linked to Increased Rates of Aggression to Non-Conspecific Targets in Male Elephants. Submitted, Under Review.

[51] Hanks J. (1972). Growth of the African Elephant (Loxodonta Africana). Afr. J. Ecol..

[52] Johnston R.E. (2008). Individual Odors and Social Communication. Advances in the Study of Behavior.

[53] Schulte B.A., Bagley K.R., Castelda S., Loizi H., Nasseri N., Vyas D.K., Goodwin T.E., East M.L., Dehnhard M. (2013). From Exploration to Selective Information Gathering: The Development of Chemosensory Investigation in Male African Elephants (Loxodonta africana). Chemical Signals in Vertebrates 12.

[54] Rasmussen L.E.L. (1995). Evidence for long-term chemical memory in elephants. Chem. Senses.

[55] Buss I.O., Rasmussen L.E., Smuts G.L. (1976). The Role of Stress and Individual Recognition in the Function of the African Elephant’s Temporal Gland. Mammalia.

[56] Sherman P.W., Reeve H.K., Pfenning D.W. (1997). Recognition systems. Behavioural Ecology.

[57] Brennan P.A., Kendrick K.M. (2006). Mammalian Social Odours: Attraction and Individual Recognition. Phil. Trans. R. Soc. B.

[58] Rasmussen L.E.L., Schulte B.A. (1998). Chemical Signals in the Reproduction of Asian (*Elephas maximus*) and African (Loxodonta Africana) Elephants. Anim. Reprod. Sci..

[59] Poole J.H., Kasman L.H., Ramsay E.C., Lasley B.L. (1984). Musth and Urinary Testosterone Concentrations in the African Elephant (Loxodonta Africana). Reproduction.

[60] Flack J.C., de Waal F. (2007). Context Modulates Signal Meaning in Primate Communication. Proc. Natl. Acad. Sci. USA.

[61] Miller M.N., Byers J.A., Bekoff M., Byers J.A. (1998). Sparring as play in young pronghorn males. Animal Play.

[62] Granweiler J., Thorley J., Rotics S. (2021). Sparring Dynamics and Individual Laterality in Male South African Giraffes. Ethology.

[63] Ganswindt A., Heistermann M., Borragan S., Hodges J.K. (2002). Assessment of Testicular Endocrine Function in Captive African Elephants by Measurement of Urinary and Fecal Androgens. Zoo Biol..

[64] Colmenares F. (1991). Greeting Behaviour between Male Baboons: Oestrous Females, Rivalry and Negotiation. Anim. Behav..

[65] Bonnie K.E., Earley R.L. (2007). Expanding the Scope for Social Information Use. Anim. Behav..

[66] Langbauer W.R. (2000). Elephant Communication. Zoo Biol..

[67] Soltis J., Leong K., Savage A. (2005). African Elephant Vocal Communication II: Rumble Variation Reflects the Individual Identity and Emotional State of Callers. Anim. Behav..

[68] De Marco A., Sanna A., Cozzolino R., Thierry B. (2014). The Function of Greetings in Male Tonkean Macaques. Am. J. Primatol..

