# Supplementary material for: Function of Trunk-Mediated “Greeting” Behaviours between Male African Elephants: Insights from Choice of Partners
Source: Animals (Basel). 2021 Sep 17;11(9):2718. doi: 10.3390/ani11092718 (PMC8467434; doi:10.3390/ani11092718)
Supplement: Supplementary file 1 [file animals-11-02718-s001.zip › animals-1373151-supplementary.pdf]

Supplementary Materials

Note S1: Definition of reciprocated trunk-SEO behaviours.

Trunk-to-SEO behaviours directed by the focal were recorded as either being one-way towards the target partner, or reciprocated back to the focal. Defining a cut-off to reciprocate a trunk-to-SEO behaviour as both 1 or 5 minutes before or after the focal contacted the target, or as being performed within the time spent continuously within one body length of the target of the behaviour, all produced identical results. Most reciprocated trunk-to-SEO behaviours occurred immediately alongside each other (personal observation).

Table S1: Output of Kruskal-Wallis H tests for each method of measuring rate of performing of trunk-to-SEO behaviours by age class of focal.

**Behavioural measure**	**Result Kruskal-Wallis H test**	
	χ^2 (3)	p
**Trunk to scent emitting organ (any of 3 organs)**		
Behaviour performed/ hour	28.697	**< 0.001**
Individuals targeted with behaviour/hour	28.009	**< 0.001**
Individuals targeted with behaviour/ potential interactor	29.517	**< 0.001**
Individuals targeted with behaviour/ potential interactor/ hour	28.913	**< 0.001**
**Trunk-to-mouth**		
Behaviour performed/ hour	26.890	**< 0.001**
Individuals targeted with behaviour/hour	26.493	**< 0.001**
Individuals targeted with behaviour/ potential interactor	27.456	**< 0.001**
Individuals targeted with behaviour/ potential interactor/ hour	26.545	**<0.001**
**Trunk-to-temporal gland**		
Behaviour performed/ hour	6.957	0.073
Individuals targeted with behaviour/hour	6.957	0.073
Individuals targeted with behaviour/ potential interactor	7.685	0.053

Individuals targeted with behaviour/ potential interactor/ hour	7.427	0.059
**Trunk-to-genitals**		
Behaviour performed/ hour	2.414	0.491
Individuals targeted with behaviour/hour	2.483	0.479
Individuals targeted with behaviour/ potential interactor	1.768	0.622
Individuals targeted with behaviour/ potential interactor/ hour	2.015	0.569

Table S2: Table of means and standard deviations of trunk-to-SEO behaviours performed by focals of different age classes during a visit to social hotspot.

	**Age class of focal**			
	10-15 years (N=50)	*16-20 years (N=63)*	*21-25 years (N=67)*	*26+ years (N=60)*
Trunk-to-SEO behaviours performed/ hour:	4.71 (4.39)	3.84 (4.27)	2.17 (2.84)	1.43 (2.17)
Individuals targeted with trunk-to-SEO behaviours/ hour:	3.15 (2.77)	2.71 (3.50)	1.56 (1.94)	1.04 (1.55)
Individuals targeted with trunk-to-SEO behaviours/ potential interactor:	0.26 (0.24)	0.27 (0.27)	0.13 (0.16)	0.10 (0.18)
Individuals targeted with trunk-to-SEO behaviours/ potential interactor/ hour:	0.34 (0.40)	0.37 (0.50)	0.16 (0.24)	0.10 (0.21)

Table S3: Post hoc pairwise comparisons using Wilcoxon rank sum test with continuity correction, showing differences between the 4 age classes concerning trunk-to-SEO behaviours performed per hour.

**Figure**	**Pairwise comparisons between age classes (Age class (years))**			
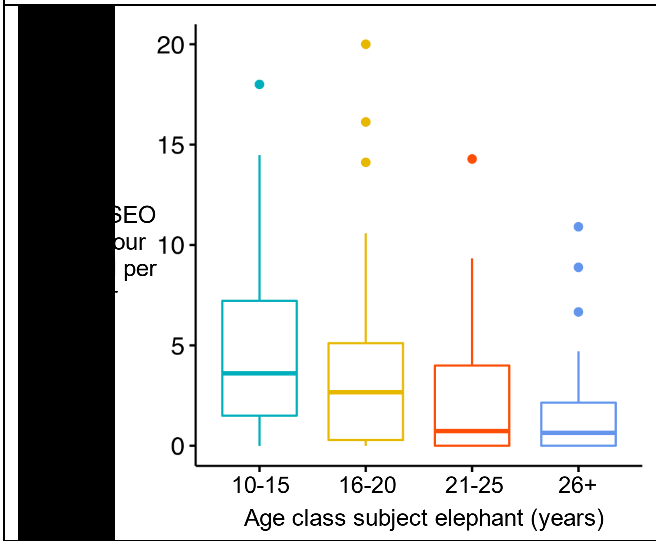		*10-15*	*16-20*	*21-25*
	16-20	0.210	-	-
	21-25	**0.001**	**0.020**	-
	26+	**<0.001**	**0.001**	0.210

P value adjustment method: BH

Both adolescent age classes performed more trunk-to-SEO behaviours of conspecifics per hour than both adult age classes

Table S4: Post hoc pairwise comparisons using Wilcoxon rank sum test with continuity correction, showing differences between the 4 age classes concerning number of individuals targeted with trunk-to-SEO behaviours per hour.

**Figure**	**Pairwise comparisons between age classes (Age class (years))**			
		10-15	*16-20*	*21-25*
	16-20	0.127	-	-
	21-25	**0.001**	**0.047**	-

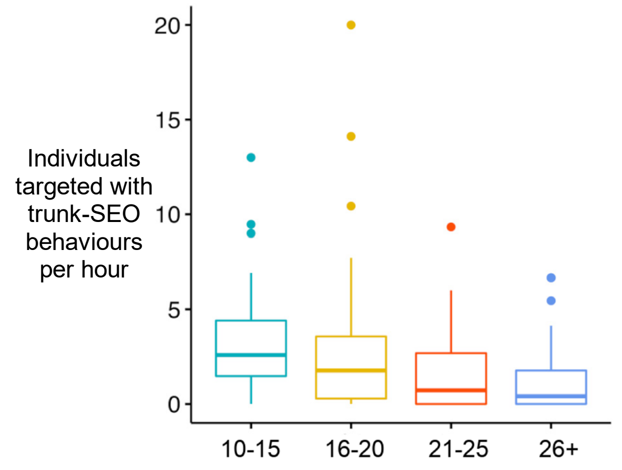

Figure			
26+	**<0.001**	**0.001**	0.167

P value adjustment method: BH

Both adolescent age classes targeted more individuals with trunk-to-SEO behaviours per hour than both adult age classes

Table S5: Post hoc pairwise comparisons using Wilcoxon rank sum test with continuity correction, showing differences between the 4 age classes concerning number of individuals targeted with trunk-to-SEO behaviours per potential interactor exposed to during visit to social hotspot.

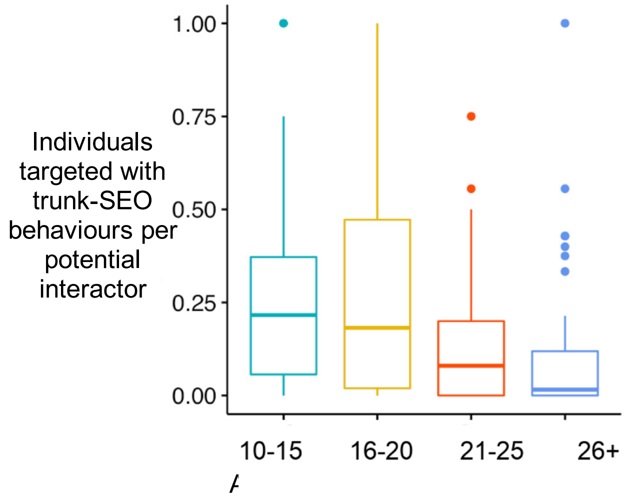

**Figure**	**Pairwise comparisons between age classes (Age class (years))**		
	10-15	*16-20*	*21-25*
16-20	0.933	-	-
21-25	**0.001**	**0.002**	-
26+	**<0.001**	**<0.001**	0.250

P value adjustment method: BH

Both adolescent age classes targeted more individuals with trunk-to-SEO behaviours per potential interactor than both adult age classes

Table S6: Post hoc pairwise comparisons using Wilcoxon rank sum test with continuity correction, showing differences between the 4 age classes concerning number of individuals targeted with trunk-to-SEO behaviours per potential interactor per hour.

**Figure**	**Pairwise comparisons between age classes (Age class (years))**			
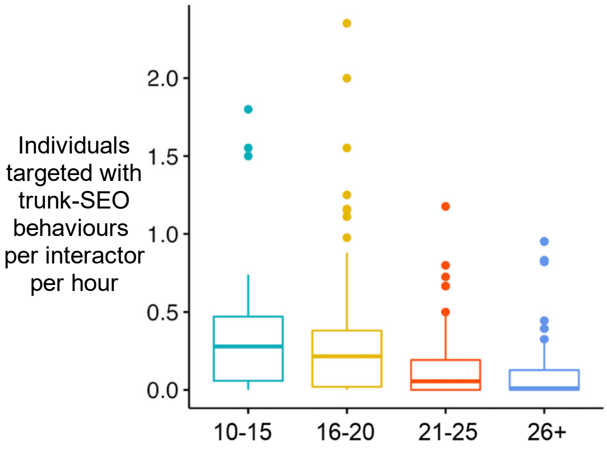		*10-15*	*16-20*	*21-25*
	16-20	0.607	-	-
	21-25	**0.002**	**0.008**	-
	26+	**<0.001**	**<0.001**	0.192

P value adjustment method: BH

Both adolescent age classes targeted more individuals with trunk-to-SEO behaviours per potential interactor per hour than both adult age classes

Table S7: Table of means and standard deviations of trunk-to-mouth behaviours performed by focals of different age classes during a visit to social hotspot.

	**Age class of focal**			
	10-15 years (N=50)	*16-20 years (N=63)*	*21-25 years (N=67)*	*26+ years (N=60)*
Trunk-to-mouth performed/ hour:	3.61 (3.79)	2.74 (3.42)	1.33 (1.75)	0.979 (1.94)
Individuals targeted with trunk-to-mouth/ hour:	2.56 (2.55)	2.10 (3.05)	1.03 (1.32)	0.726 (1.27)

Individuals targeted with trunk-to-mouth/ potential interactor:	0.221 (0.234)	0.212 (0.251)	0.086 (0.111)	0.066 (0.116)
Individuals targeted with trunk-to-mouth/ potential interactor/ hour:	0.297 (0.366)	0.293 (0.416)	0.112 (0.197)	0.072 (0.161)

Table S8: Pairwise comparisons using Wilcoxon rank sum test with continuity correction, showing differences between the 4 age classes concerning trunk-to-mouth behaviours performed per hour.

**Pairwise comparisons between age classes (Age class (years))**			
	10-15	*16-20*	*21-25*
16-20	0.255	-	-
21-25	**0.001**	**0.017**	-
26+	**<0.001**	**<0.001**	0.156

P value adjustment method: BH

Both adolescent age classes directed more trunk-to-mouth behaviours to conspecifics per hour than both adult age classes

Table S9: Post hoc pairwise comparisons using Wilcoxon rank sum test with continuity correction, showing differences between the 4 age classes concerning number of individuals targeted with trunk-to-mouth behaviours per hour.

**Pairwise comparisons between age classes (Age class (years))**			
	10-15	*16-20*	*21-25*
16-20	0.173	-	-
21-25	**0.001**	**0.264**	-
26+	**<0.001**	**<0.001**	0.173

P value adjustment method: BH

Both adolescent age classes targeted more individuals with trunk-to-mouth behaviours per hour than both adult age classes

Table S10: Post hoc pairwise comparisons using Wilcoxon rank sum test with continuity correction, showing differences between the 4 age classes concerning number of individuals targeted with trunk-to-mouth behaviours per potential interactor exposed to during visit to social hotspot.

**Pairwise comparisons between age classes (Age class (years))**			
	10-15	*16-20*	*21-25*
16-20	0.734	-	-
21-25	**0.002**	**0.003**	-
26+	**<0.001**	**<0.001**	0.245

P value adjustment method: BH

Both adolescent age classes targeted more individuals with trunk-to-mouth behaviours per potential interactor than both adult age classes

Table S11: Post hoc pairwise comparisons using Wilcoxon rank sum test with continuity correction, showing differences between the 4 age classes concerning number of individuals targeted with trunk-to-mouth behaviours per potential interactor per hour.

**Pairwise comparisons between age classes (Age class (years))**			
	10-15	*16-20*	*21-25*
16-20	0.578	-	-
21-25	**0.002**	**0.008**	-
26+	**<0.001**	**<0.001**	0.213

P value adjustment method: BH

Both adolescent age classes targeted more individuals with trunk-to-mouth behaviours per potential interactor per hour than both adult age classes

Table S12: Table of means and standard deviations of trunk-to-temporal-gland behaviours performed by focals of different age classes during a visit to social hotspot.

	Age class of focal			
	10-15 years (N=50)	*16-20 years (N=63)*	*21-25 years (N=67)*	*26+ years (N=60)*
Trunk-to-temporal-gland performed/ hour:	0.200 (0.603)	0.502 (1.030)	0.389 (0.868)	0.197 (0.523)
Individuals targeted with trunk-to-temporal-gland/ hour:	0.200 (0.603)	0.502 (1.030)	0.389 (0.868)	0.197 (0.523)
Individuals targeted with trunk-to-temporal-gland/ potential interactor:	0.015 (0.044)	0.066 (0.145)	0.029 (0.066)	0.021 (0.064)
Individuals targeted with trunk-to-temporal-gland/ potential interactor/ hour:	0.016 (0.049)	0.061 (0.159)	0.030 (0.076)	0.018 (0.064)

Table S13: Table of means and standard deviations of trunk-to-genitals behaviours performed by focals of different age classes during a visit to social hotspot.

	Age class of focal			
	10-15 years (N=50)	*16-20 years (N=63)*	*21-25 years (N=67)*	*26+ years (N=60)*
Trunk-to-genitals performed/ hour:	0.677 (1.20)	0.496 (1.17)	0.454 (0.12)	0.238 (0.526)
Individuals targeted with trunk-to-genitals/ hour:	0.617 (1.08)	0.465 (1.13)	0.381 (0.928)	0.227 (0.508)

Individuals targeted with trunk-to-genitals/ potential interactor:	0.048 (0.090)	0.047 (0.120)	0.027 (0.064)	0.034 (0.091)
Individuals targeted with trunk-to-genitals/ potential interactor/ hour:	0.079 (0.180)	0.076 (0.237)	0.037 (0.098)	0.030 (0.089)

Probability of directing trunk-SEO behaviour in 10-min follow

age (years)

15

20

25

Number of other elephants present at hotspot

Figure S1: The number of other elephants present with the focal did not significantly effect probability of directing a trunk-to-SEO behaviour in a 10-minute follow. (GLMM of "trunk-to-SEO behaviour directed in 10-min follow" predicted by interaction of focal age class and "Number of other elephants present with focal at hotspot": Regression coefficients, adjusted odds ratios and 95% confidence intervals of "Number of other elephants present with focal at hotspot": 10-15 as reference class: 0.017, 1.017 (0.972-1.064), p = 0.462; 16-20 as reference class: 0.043, 1.044 (0.991-1.101), p = 0.104; 21-25 as reference class: 0.043, 1.044 (0.990-1.102), p = 0.115; 26+ as reference class: 0.043, 1.044 (0.982-1.109), p = 0.169.

Table S14 GLMM output of likelihood of a trunk-to-SEO behaviour being a reciprocated event, predicted by age class of the focal subject.

**Reference class**	**Predictor**	**Coefficient**	**aOR (+95% CI)**	**P**
10 – 15 years	16 – 20 years	0.579	1.785 (1.070 - 2.977)	**0.026**
	21 – 25 years	1.127	3.088 (1.750 - 5.446)	**< 0.001**
	26 + years	1.002	2.722 (1.470 - 5.041)	**0.001**
16 – 20 years	10 – 15 years	-0.579	0.560 (0.336 - 0.934)	**0.027**
	21 – 25 years	0.548	1.730 (1.022 - 2.927)	**0.041**
	26 + years	0.422	1.525 (0.852 - 2.730)	0.155
21 – 25 years	10 – 15 years	-1.127	0.324 (0.184 - 0.571)	**<0.001**
	16 – 20 years	-0.548	0.578 (0.342 - 0.978)	**0.041**
	26 + years	-0.126	0.882 (0.473 - 1.643)	0.692
26 + years	10 - 15 years	-1.002	0.367 (0.198 - 0.680)	**0.001**
	16 - 20 years	-0.422	0.656 (0.366 - 1.174)	0.155
	21 - 25 years	0.126	1.134 (0.609 - 2.113)	0.692

aOR= adjusted odds ratio, CI = confidence intervals

The age class of the focal subject predicted whether a trunk-to-SEO behaviour was a reciprocated, as opposed to a one-way event. 10 – 15-year-olds had a lower probability of a directed trunk-to-SEO behaviour being a reciprocated event than all other age classes. 16 – 20-year-olds had a higher probability of being in a reciprocated trunk-to-SEO event than 10-15 year olds, but lower probability than 21-25 year olds. 21 – 25-year-olds and 26+ years olds did not differ from one another concerning whether their trunk-to-SEO behaviour were reciprocated events or not.

Table S15: Observed odds ratios and permutation based significances of elephants targeting an age-matched individual relative to non-age-matched individual with trunk-to-SEO behaviours of different target organs.

**Behaviour directed to conspecific**	**Observed odds ratio of directing behaviour to age-matched relative to non-age-matched individual**	**95% CI Randomised odds ratios**	**P**
Trunk-to-SEO behaviour (all organs combined)	2.275	0.887-1.419	**<0.001**
Trunk-to-mouth	2.374	0.980-1.646	**<0.001**
Trunk-to-temporal gland	1.569	0.632-2.037	0.373
Trunk-to-genitals	2.390	0.580-2.000	**0.012**

CI = confidence intervals

Table S16: Observed adjusted odds ratios and permutation based significances of elephants of different age class targeting an age-matched relative to non-age-matched individual with trunk-to-SEO behaviours of different target organs.

**Behaviour directed to conspecific**	**Age class (years)**	**Observed odds ratio of directing behaviour to age-matched relative to non age-matched individual**	**95% CI randomised odds ratios**	**P**
**Trunk-to-SEO behaviour (all organs combined)**	10-15	3.268	0.818-1.775	**< 0.001**
	16-20	1.454	0.700-1.508	0.085
	21-25	2.056	0.445-1.730	**0.014**
	26+	2.185	0.232-1.912	**0.048**
**Trunk-to-mouth**	10-15	3.389	0.991-2.208	**< 0.001**
	16-20	1.532	0.691-1.607	0.077
	21-25	1.905	0.366-2.190	0.134
	26+	2.228	0.375-2.242	0.061
**Trunk-to-temporal-gland**	10-15	0.340	0.243^{-6}-5.342	0.233
	16-20	1.729	0.486-2.564	0.317
	21-25	1.671	0.110^{-6}-2.373	0.400
	26+	2.217	0.191^{-6}-3.756	0.581
**Trunk-to-genitals**	10-15	4.042	0.499-4.058	0.069
	16-20	1.102	0.237-2.271	0.789
	21-25	4.078	0.551^{-7}-2.717	**0.026**
	26+	0.758	0.965^{-7}-3.945	0.713

CI = confidence intervals

Table S17: Observed odds ratios and permutation based significances of elephants targeting an elephant aged 26+ years relative to a younger male with trunk-to-SEO behaviours of different target organs.

Behaviour directed to conspecific	Observed adjusted odds ratio of directing behaviour to elephant aged 26+ years relative younger male	95% CI randomised odds ratios	P
Trunk-to-SEO behaviour (all 3 target organs combined)	1.276	0.688-1.512	0.322
Trunk-to-mouth	1.327	0.646-1.532	0.283
Trunk-to-temporal gland	0.970	0.431-3.066	0.614
Trunk-to-genitals	1.560	0.265-2.078	0.208

CI = confidence intervals

Table S18: Observed adjusted odds ratios and permutation based significances of elephants of different age classes targeting a male aged 26+ years relative to younger male with trunk-to-SEO behaviours of different target organs.

**Behaviour directed to conspecific**	**Age class (years)**	**Observed adjusted odds ratio of directing behaviour to 26+ year old relative to younger elephant**	**95% CI Randomised odds ratios**	**P**
**Trunk-to-SEO behaviour (all organs combined)**	10-15	0.816	0.416-1.926	0.574
	16-20	1.081	0.503-2.045	0.839
	21-25	1.668	0.459-2.425	0.455
	26+	2.077	0.251-2.074	**0.042**
**Trunk-to-mouth**	10-15	0.794	0.275-2.394	0.487
	16-20	1.250	0.382-2.244	0.814
	21-25	1.788	0.506-2.985	0.572
	26+	2.052	0.196-2.053	0.052
**Trunk-to-temporal-gland**	10-15	0.941^{-7}	0.926^{-7}-5.784	0.171
	16-20	0.804	0.456^{-7}-7.01	0.478
	21-25	0.123^{-6}	0.122^{-6}-15.640	0.223
	26+	2.405	0.702^{-7}-4.070	0.583
**Trunk-to-genitals**	10-15	1.448	0.102^{-6}-2.584	0.675
	16-20	2.076	0.231^{-6}-4.868	0.512
	21-25	1.928	0.443^{-7}-3.011	0.340
	26+	0.810	0.944^{-7}-4.234	0.921

CI = confidence intervals

Only 10-minute focal follows where at least one elephant of each age category (26+ and younger than 26 years) was present as a potential interactor were included in models. In the case of trunk-to-temporal-gland behaviours, despite a total of 456 follows being included in these models, only 23 trunk-to-temporal-gland behaviours were observed in these follows, including 0 trunk-to-temporal-gland behaviours towards 26+ year olds from elephants aged 10-15 years and 21-25 years, and only 1 trunk-to-temporal-gland behaviour of a 26+ year old by 16-20 year olds. Similarly the trunk-to-genital model was generated from only 40 observed trunk-to-genital behaviours, 9 of which were to elephants aged 26+ years. The small number of trunk behaviours in both these models may explain the wide randomised confidence intervals, and a larger data set may be warranted (despite 456 follows equivaling 76 study hours of elephant behaviour) to explore the

observed trends for lower odds of elephants younger than 26+ years directing trunk-to-temporal-gland behaviours to mature males over 26 years relative to younger male potential interactors, and higher odds of elephants younger than 26+ years directing trunk-to-genital behaviours to elephants over 26 years relative to younger male potential interactors.

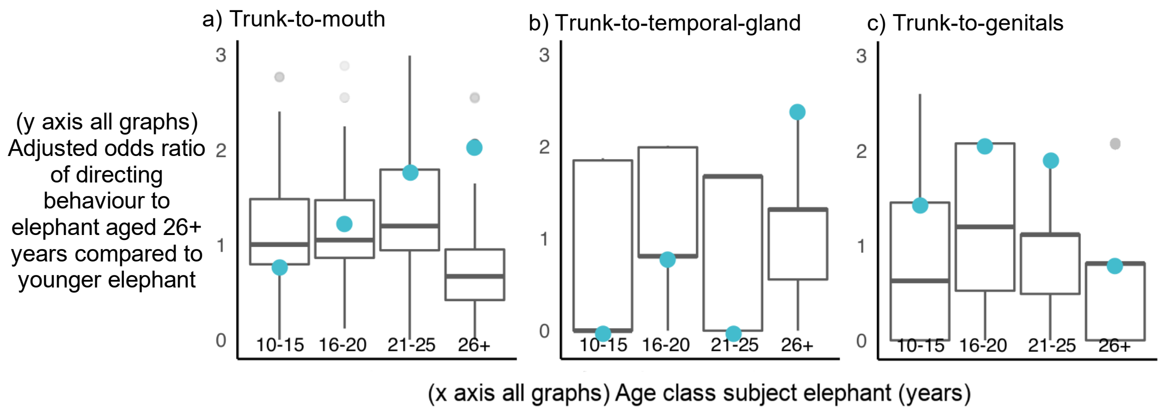

Figure S2: Observed adjusted odds ratios of elephants directing trunk-to-SEO behaviours to an elephant aged 26+ years relative to a younger elephant (blue circles), plotted against randomly permuted adjusted odds ratios of directing trunk-to-SEO behaviour to elephant aged 26+ years relative to a younger elephant (boxplots with median, interquartile range, minimum and maximum values). All age classes directed trunk behaviours to the mouth, temporal glands and genitals of elephants aged 26+ years as expected by random chance (Supplementary table S18 for observed adjusted odds ratios, 95% confidence intervals and p values for each age class and target organ).

Table S19: GLMM outputs of likelihood of elephant directing trunk-to-SEO behaviours to conspecific during a 10-minute focal follow by different time conditions during his visit to social hotspots

**Predictor**	**Coefficient**	**aOR (+95% CI)**	***P* value**
Intercept	-1.202	0.301 (0.175-0.516)	**<0.001**

% Time progression within total focal follow (Figure S3)		< - 0.001	1 (0.993-1.009)	0.815
Is first 10-minutes of full focal follow	No	*Ref*	*Ref*	
	Yes	-0.510	0.600 (0.346-1.041)	0.069
Is last 10-minutes of full focal follow	No	*Ref*	*Ref*	
	Yes	-0.385	0.680 (0.380-1.217)	0.194

aOR= adjusted odds ratio, CI = confidence intervals

The percent of time progressed within a focal follow that a particular 10-minute follow occupied did not predict likelihood of a trunk-to-SEO behaviour being performed by a focal. There was no difference between likelihood of focals directing trunk-to-SEO behaviours to conspecifics in their last 10-minutes at hotspots, compared to earlier 10-minute follows, nor in their first 10-minutes at hotspots compared to later follows. Focal ID included as random effect.

Probability of directing a trunk-SEO behaviour to conspecific in 10-min follow

Percent progressed in full focal follow

Figure S3: There was no change in a focals probability of directing a trunk-to-SEO behaviour to a conspecific over the course of his stay at a focal hotspot. The percent of time progressed within a focal follow that a particular 10-minute follow occupied did not predict likelihood of

trunk-to-SEO behaviours being performed by focals (aOR (95% CI)= 1 (0.993-1.009), p = 0.815).

Table S20: Observed odds ratios and permutation-based significances of elephants targeting an elephant met new at the river relative to elephant arrived at river with, with trunk-to-SEO behaviours of different target organs.

**Behaviour directed to conspecific**	**Observed odds ratio of directing behaviour to new elephant relative to elephant arrived at river in a group with**	**95% CI randomised odds ratios**	**P**
Trunk-to-SEO behaviours (all 3 target organs combined)	0.711	0.642-1.457	0.182
Trunk-to-mouth	0.693	0.621-1.500	0.145
Trunk-to-temporal-gland	0.770	0.472-2.141	0.521
Trunk-to-genitals	0.929	0.477-2.185	0.947

CI = confidence intervals

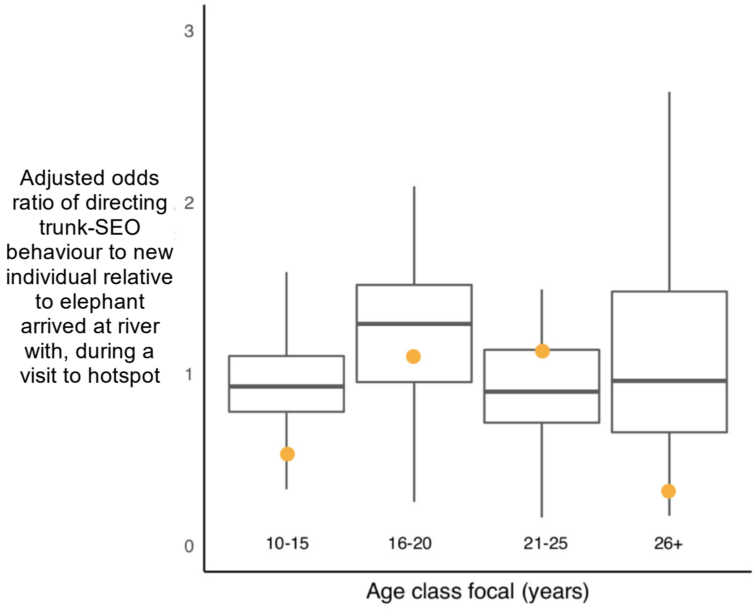

Figure S4: Observed adjusted odds ratios of subjects directing trunk-to-SEO behaviours to an individual met new at the hotspot relative to an elephant he arrived at the river with (orange

circles), plotted against permuted adjusted odds ratios of directing trunk-to-SEO behaviours to new elephants relative to elephants arrived with at hotspots (boxplots with median, interquartile range, minimum and maximum values). All age classes of focal directed trunk-to-SEO behaviours to new individuals within the range expected by random assignment of behaviours to all elephants present (observed odds ratios for ages: 10-15 years = 0.560, 95% CI random = (0.472-1.939), $p = 0.175$; 16-20 years = 1.110, 95% CI random = (0.600-2.472), $p = 0.727$; 21-25 years = 1.138, 95% CI random = (0.384-2.676), $p = 0.625$; 26+ years = 0.340, 95% CI random = (0.339-2.580), $p = 0.059$).
